# The melibiose-derived glycation product mimics a unique epitope present in human and animal tissues

**DOI:** 10.1038/s41598-021-82585-7

**Published:** 2021-02-03

**Authors:** Magdalena Staniszewska, Agnieszka Bronowicka-Szydełko, Kinga Gostomska-Pampuch, Jerzy Szkudlarek, Arkadiusz Bartyś, Tadeusz Bieg, Elżbieta Gamian, Agata Kochman, Bolesław Picur, Jadwiga Pietkiewicz, Piotr Kuropka, Wiesław Szeja, Jerzy Wiśniewski, Piotr Ziółkowski, Andrzej Gamian

**Affiliations:** 1 Hirszfeld Institute of Immunology and Experimental Therapy, Polish Academy of Sciences, Weigla 12, 53-114 Wrocław, Poland; 2 Centre for Interdisciplinary Research, The John Paul II Catholic University of Lublin, Konstantynow 1J, 20-708 Lublin, Poland; 3 Department of Medical Biochemistry, Wroclaw Medical University, Chalubinskiego 10, 50-368 Wrocław, Poland; 4 Department of Organic Chemistry, Bioorganic Chemistry and Biotechnology, Silesian University of Technology, Krzywoustego 4, 44-100 Gliwice, Poland; 5 Department of Pathomorphology, Wroclaw Medical University, Marcinkowskiego 1, 50-368 Wrocław, Poland; 6 Department of Pathology, University Hospital Monklands, Monkscourt Ave, Airdrie, ML6 0JS UK; 7 Faculty of Chemistry, University of Wrocław, 50-383 Wrocław, Poland; 8 Department of Anatomy and Histology, Wroclaw University of Environmental and Life Sciences, Norwida 1, 50-375 Wrocław, Poland; 9 Wroclaw Research Centre EIT+, PORT, Stabłowicka 147/149, 54-066 Wrocław, Poland

**Keywords:** Biochemistry, Biotechnology, Diseases, Molecular medicine

## Abstract

Non-enzymatic modification of proteins by carbohydrates, known as glycation, leads to generation of advanced glycation end-products (AGEs). In our study we used in vitro generated AGEs to model glycation in vivo. We discovered in vivo analogs of unusual melibiose-adducts designated MAGEs (mel-derived AGEs) synthesized in vitro under anhydrous conditions with bovine serum albumin and myoglobin. Using nuclear magnetic resonance spectroscopy we have identified MAGEs as a set of isomers, with open-chain and cyclic structures, of the fructosamine moiety. We generated a mouse anti-MAGE monoclonal antibody and show for the first time that the native and previously undescribed analogous glycation product exists in living organisms and is naturally present in tissues of both invertebrates and vertebrates, including humans. We also report MAGE cross-reactive auto-antibodies in patients with diabetes. We anticipate our approach for modeling glycation in vivo will be a foundational methodology in cell biology. Further studies relevant to the discovery of MAGE may contribute to clarifying disease mechanisms and to the development of novel therapeutic options for diabetic complications, neuropathology, and cancer.

## Introduction

Glycation has attracted extensive scientific interest for its role in the pathology of common diseases such as diabetes and age-related disorders. Non-enzymatic reaction between proteins and reducing sugars or other aldehydes, followed by a series of chemical rearrangements, results in the formation of advanced glycation end-products (AGEs)^1,2^. The diversity of possible glycation substrates (sugars, ascorbic acid, lipid oxidation metabolites and nucleotides) and the complexity of glycation pathways results in AGEs of various structures and physicochemical properties. The list of glycation adducts is far from complete, thus our understanding of the properties and role of AGEs in cell biology remains largely unelucidated. To date only a small percentage of AGEs have been structurally characterized^3^, the best known of which are carboxymethyllysine (CML), pentosidine, and argpyrimidine^4,5^. However, these well-characterized structures constitute only a minor fraction of the entire pool of AGEs. Products formed during food thermal processing can be delivered to an organism via diet, are absorbed in the intestines^6^, and so constitute the exogenous source of glycation adducts^3^. Excess AGEs are partially cleared from the blood hepatically and are renally excreted^7^. As effective clearance declines with age^7^, a substantial amount of the ingested AGEs is retained in the organism^8^. AGEs form also in vivo and extensive protein modification by AGEs has been observed in several tissues during aging and diabetes^9,10^ and contributes to cataract formation^4^, Alzheimer disease^11,12^, atherosclerosis^13^ and cancer^14^. Extracellular matrix proteins are especially susceptible to AGE accumulation due to their low turnover rate and the resistance of the glycated proteins to proteolysis^15,16^. AGE accumulation contributes to stiffening of arteries and heart muscle^17,18^ and impairs vascular repair^19^.

The structural heterogeneity and diverse effects of AGEs compel further research on the structure and biological role of common AGEs that accumulate in human tissues. Obtaining model AGEs, which mimic natural analogs, is critical for such studies and allows for the development of diagnostic assays, i.e. immunochemical methods^20–22^. Glucose (glc), ribose (rib), methylglyoxal (MGO) and few others are the most often used carbohydrates for in vitro AGE preparation^21,23,24^. However, none of the resulting products can universally represent the glycation process in tissue. Here, we use high-pressure (HPG) and high temperature glycation (HTG) to generate model glycation products^21^ in vitro. We further show that an unusual carbohydrate—melibiose (α-D-gal-(1 → 6)-D-glc), delivered to the human organism mostly through a plant diet^25^, honey^26^ or provided by gut microbiota^27^—generates in the in vitro reaction a MAGE product that resembles the most common adduct we found to be present in several tissues of humans and numerous animal species. The tissue native counterpart of MAGE is expected to play a significant role in animal biology.

## Results

**Figure 1:**
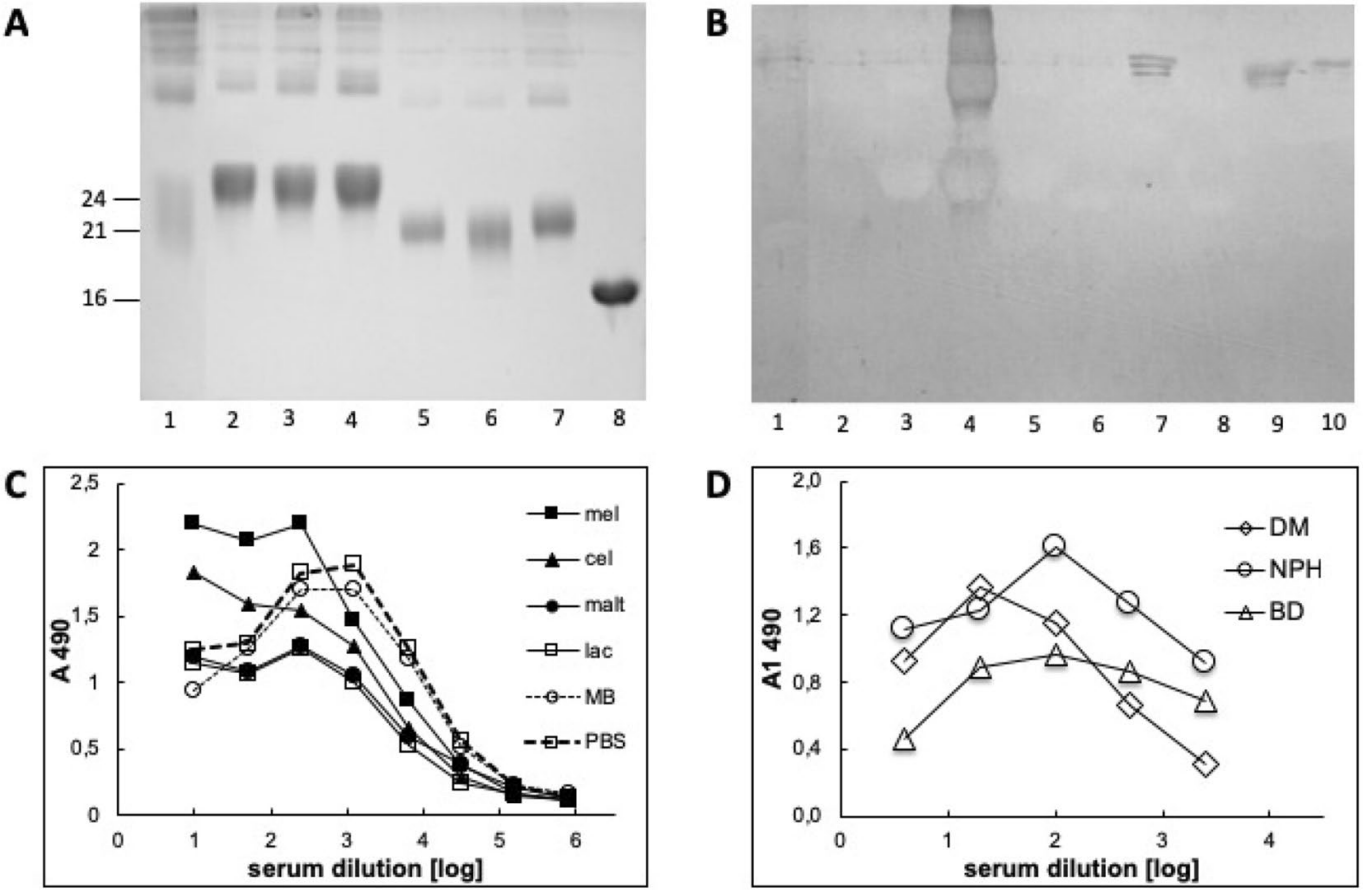
Autoantibodies present in human serum bind different model AGEs. (**A**) 12.5% SDS-PAGE separation of MB (lane 8) and its glycation products obtained in HTG reaction with: lac (lane 1), mal (lane 2), cel (lane 3), mel (lane 4), glc (lane 5), man (lane 6), gal (lane 7). Molecular mass is indicated with lines on the left side of the picture. (**B**) WB of serum from diabetic patient with control unmodified protein MB (lane 8), BSA (lane 9), and model AGEs: MB-lac (lane 1), MB-mal (lane 2), MB-cel (lane 3), MB-mel (lane 4), MB-glc (lane 5), MB-man (lane 6), MB-gal (lane 7), BSA-lac (lane 10) formed in HTG or HPG conditions (lane 1–7 and 10, respectively). (**C**) ELISA of serum from diabetic patients on a plate coated with unmodified MB (open circle) or MB glycated by different carbohydrates (filled marks); the control of secondary Ab were evaluated on wells with BSA as a blocking agent (open square); values presented are of serum from a representative patient; (**D**) ELISA with representative sera from diabetic (DM), diabetic nephropathy (NPH) and Buerger’s disease (BD) patients on plate coated with MB-mel; the results (A_1_ 490) were normalized by subtraction of the absorption of secondary Ab (PBS in place of serum).

We applied dry conditions under high temperature (HTG) and aqueous conditions under high pressure (HPG) to generate AGEs with distinct structural properties in contrast to the conventional reaction carried out in water solution under ambient pressure (aqueous conventional glycation—ACG)^21^. A series of model AGEs on myoglobin (MB) or bovine serum albumin (BSA) were generated with a variety of mono- and disaccharides, including glucose (glc), galactose (gal), fructose (fru), mannose (man), lactose (lac), maltose (mal), melibiose (mel), and cellobiose (cel). The products formed with these proteins from disaccharides had a higher molecular mass (Fig. 1A, lanes 1–4) than products formed from monosaccharides (Fig. 1A, lanes 5–7) as shown by electrophoresis on polyacrylamide gel.

Since AGE-modified proteins induce production of autoantibodies in pathological conditions^28–31^, we hypothesized that structurally different AGEs can induce distinct autoantibodies. In the literature, glucose is considered the major substrate for glycation products in serum^12,32,33^ thus we tested human serum (containing a pool of antibodies) for binding to a variety of model HTG-, HPG- and ACG-generated in vitro AGEs. Several diabetic patients were screened by Western blotting, which confirmed the presence of autoantibodies reacting with model AGEs generated from multiple precursors, including mel and gal (Fig. 1B, lane 4, 7). While glc HTG derivatives were not recognized by human autoantibodies, intense binding was observed for a product formed from the disaccharide mel (mel-derived AGEs, MAGEs). The same reaction pattern of human serum was observed by ELISA (Fig. 1C). Autoantibodies specific to MAGEs were also present in sera of patients with other conditions, i.e. Buerger’s disease (Fig. 1D). These results suggest that the AGEs generated from glc under HTG conditions, might be minor antigenic products. These findings prompted us to further investigate the properties and structure of MAGEs.

**Figure 2:**
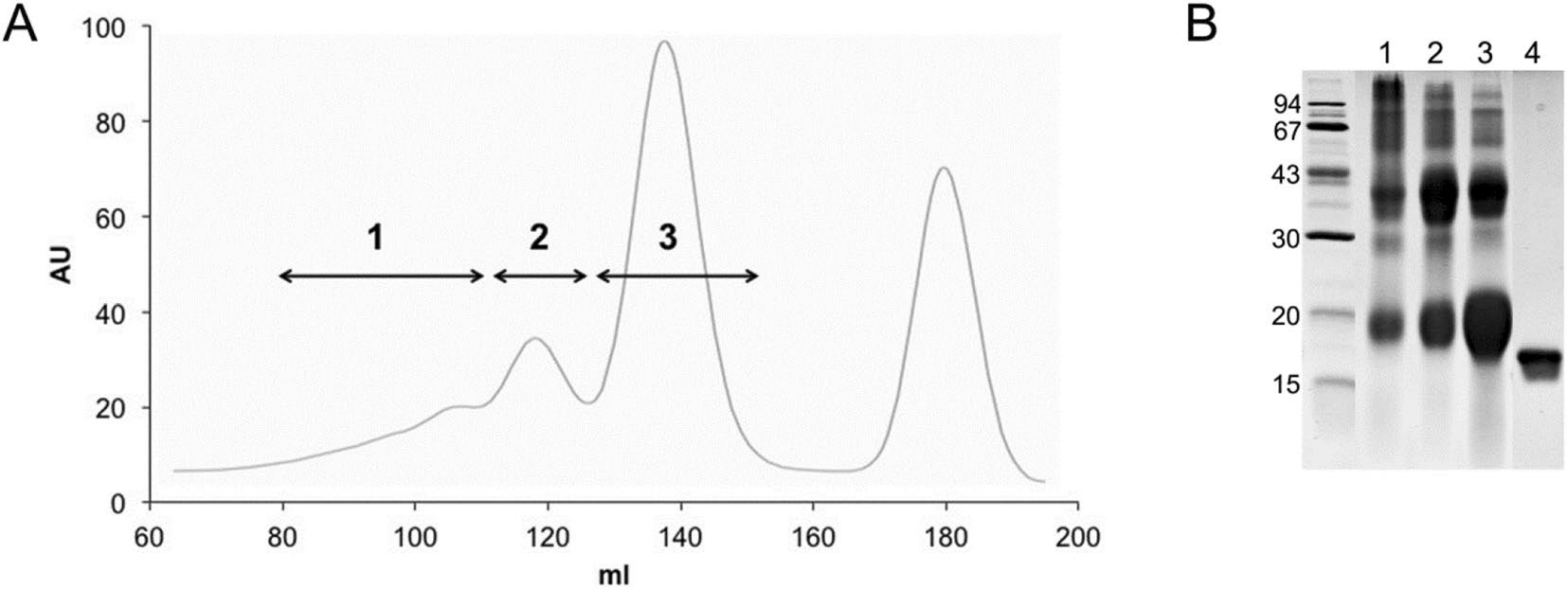
Fractionation of products obtained in HTG reaction of MB with mel (MAGEs fr. 1–3). (**A**) typical elution profile from a Sephadex G-200 column: tubes with eluted material were pooled as fractions 1–3 for analysis while low molecular mass material was ignored. (**B**) Products present in fraction 1, 2, and 3 (lane 1–3) and MB used for glycation (lane 4) were analyzed on 12.5% SDS-PAGE gel. The molecular mass of the reference standards is indicated on the left.

We glycated MB under HTG conditions by heating the lyophilized mixture of protein and mel as described in “Methods” using an oven or microwave reactor that facilitates more stable and consistent reaction conditions (MWG—microwave glycation). The obtained mixture of products was fractionated on a Sephadex G-200 column (Fig. 2A) into high-molecular mass material (fractions 1 and 2), glycated protein monomer (fraction 3), and unbound mel with yields of 16.5%, 22.3% and 61.2% for fractions 1, 2, and 3, respectively (Table 1). The main product present in MAGE fr. 3 was the glycated protein monomer (Fig. 2B, Table 1). The GLC-MS sugar analysis of fr. 1 and 3 showed the presence of gal while glc was not detected, indicating that this material constitutes AGEs with terminal gal derived from the bound mel. Interestingly, the products in fr. 2 represented proteins with attached, modified mel that released only a low amount of intact gal during hydrolysis. This suggested a rearrangement of the bound α-D-gal-(1 → 6)-D-glc. We also recorded characteristic AGE fluorescence emission (λ_em_ 440 nm) after excitation with light at λ_ex_ 370 nm^34^*.* Emission intensities (Table 1) for the cross-linked material (fr. 1 and 2) were higher than for the monomeric (fr. 3) products.

**Table 1:** Analysis of the products present in fractions after separation of MAGE (MB-mel) on a Sephadex G-200 column.

Sample	Mean yield (%)^a^	Molecular mass (kDa)^b^	Gal/Glc^c^	Fluorescence (IFU)^d^
MAGE fr.1	16.5 ± 5.6	> 42	12.6	13.819
MAGE fr.2	22.3 ± 6.4	42	0.7	7.57
MAGE fr.3	61.2 ± 3.9	21 (17.155)	10.2	3.924
MB	–	17 (16.952)	0.2	4.032

^a^Calculated as the percentage of total weight of the collected material; mean of 5 experiments.

^b^Counted from the migration in SDS–polyacrylamide gel; in parentheses values from MALDI-MS spectra are given.

^c^Measured using GLC-MS method.

^d^Intensity of fluorescence emission at 440 nm after excitation with a wavelength of 370 nm, expressed as the arbitrary intensity fluorescence units [IFU] per 0.5 mg protein from a representative experiment. Each value is averaged from three scans.

**Figure 3:**
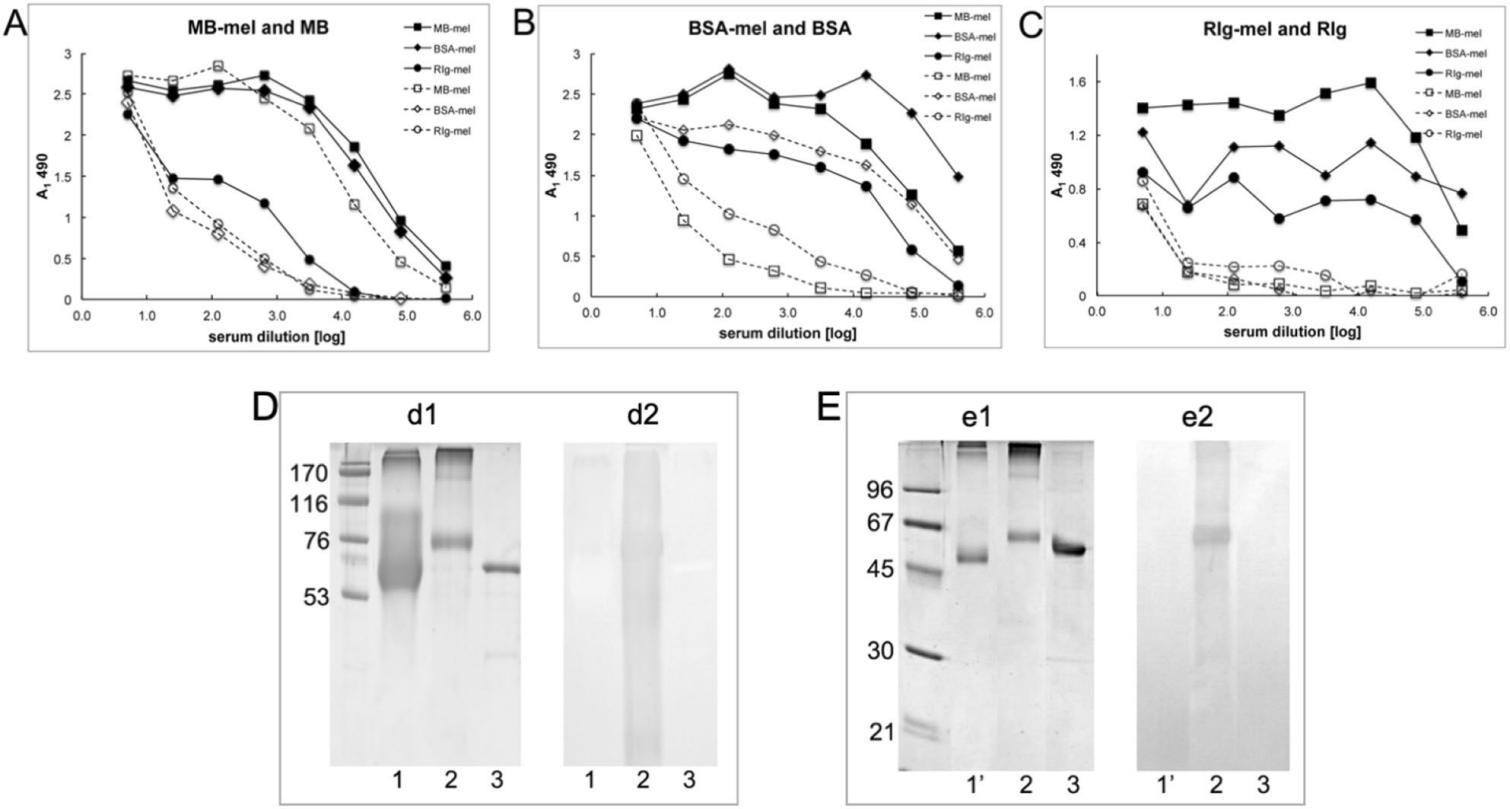
Characterization of the polyclonal anti-MAGE antisera. ELISA of anti-MB-mel, anti-BSA-mel, and anti-RIg-mel rabbit sera on plates coated with (**A**) MB-mel (solid lines) or MB (dashed lines); (**B**) BSA-mel (solid lines) or BSA (dashed lines); (**C**) RIg-mel (solid lines) or RIg (dashed lines); the results were normalized by subtraction of the absorption of secondary Ab (PBS in place of serum) and expressed as A_1_ 490. The glycation products generated from melibiose show different antigenic properties than products formed from (**D**) glycolaldehyde (GA) and (**E**) methylglyoxal (MGO). The samples were as follows: (1) BSA-GA (24 µg), (1′) BSA-MGO (2 µg), (2) BSA-mel (1 µg), (3) BSA (2 µg) were resolved in 10% polyacrylamide gel stained with CBB (**d1**,**e1**) or transferred onto PVDF membrane for Western blotting (**d2**,**e2**) with the anti-MAGE (MB-mel) rabbit serum.

In order to determine whether MAGEs form on different proteins and to compare their structural properties we employed specific sera raised against MAGEs and developed immunochemical assays. A panel of rabbit sera against the cross-linked products of rabbit immunoglobulin (RIg), bovine serum albumin (BSA), and MB were generated and used for ELISA on plates coated with the cross-linked MAGEs of corresponding carrier proteins. The results showed cross-reactivity of MAGEs formed on all used proteins with different anti-MAGE antibodies (Fig. 3A–C) and suggest that synthesized MAGEs display a common antigenic structure independent of the carrier protein and differ from other common glycation products formed by reaction of BSA with GA or MGO (Fig. 3D,E).

**Figure 4:**
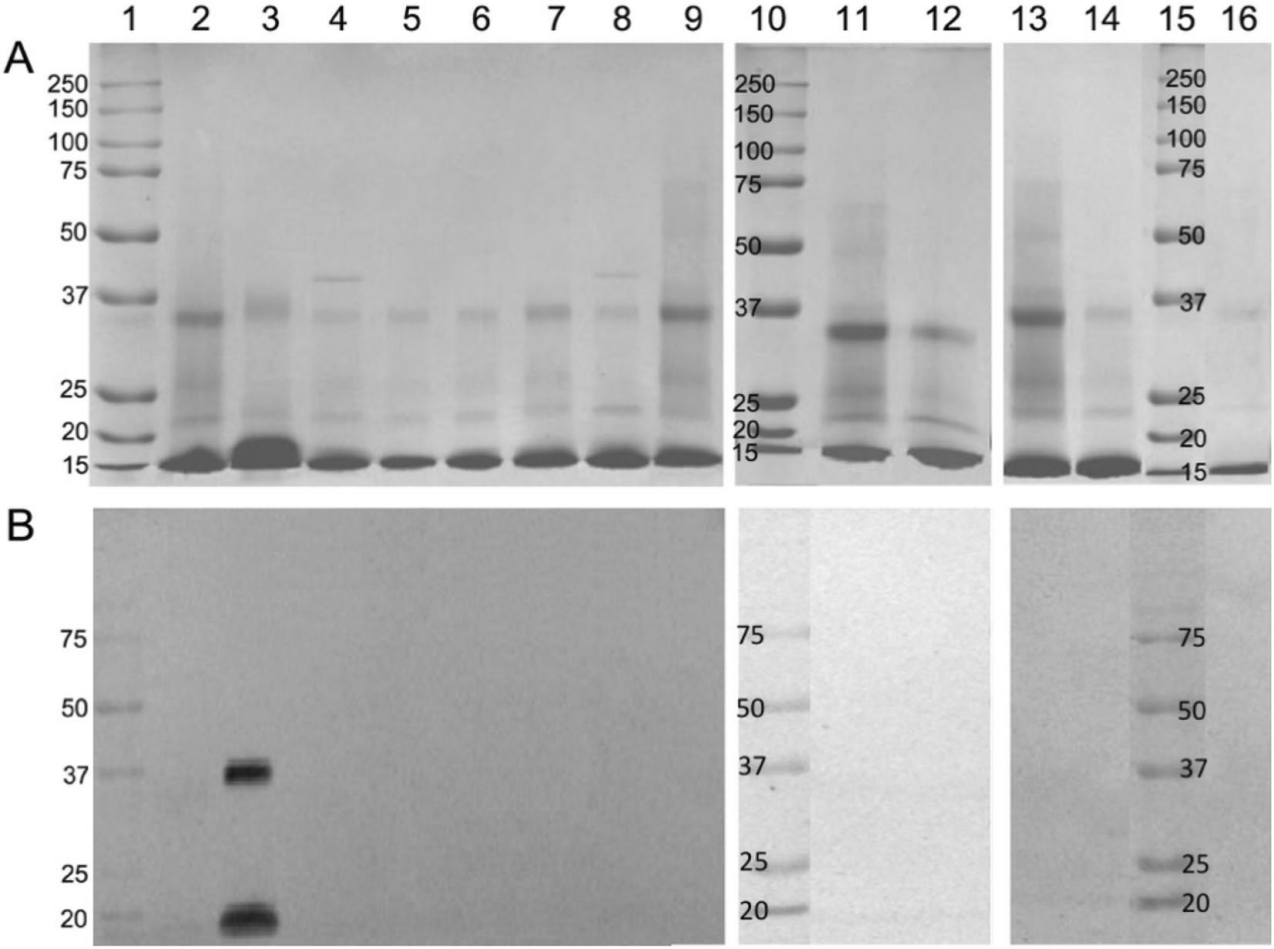
Reactivity of anti-MAGE mAb with the model glycation products. The MB (lane 16) was incubated in solution (lanes 2, 4, 6, 8, 11, 13) or in dry conditions (3, 5, 7, 9, 12, 14) with mel (2, 3), lac (4, 5), fru (6, 7), glc (8, 9), MGO (11, 12), GA (13, 14). The protein glycation products (50 µg/well) separated on 12.5% SDS-PAGE gel were stained with Coomassie Brilliant Blue (**A**) or transferred onto the membrane probed with the anti-MAGE/10 mAb (**B**).

Further analysis of MAGE properties was carried out with affinity purified rabbit anti-MAGE serum on MAGE, MB and mel-coupled columns, respectively (Supplementary Fig. S1). We thus obtained a pure fraction, deprived of anti-MB and anti-mel Abs, which we designated rabbit anti-MAGE Abs. Reactivity of the purified Abs was tested by Western blotting and ELISA on plates coated with MB-mel (Supplementary Fig. S2A), in which assays these Abs recognized MAGEs independently of the carrier protein, i.e. cross-linked products of BSA-mel (Supplementary Fig. S2B, lanes 1, 2, 3). Binding of antibodies was unique for AGEs derived from mel under HTG conditions (Supplementary Fig. S2C, lane 5) in comparison to AGEs generated during an HPG reaction or products formed from fru or lac (Supplementary Fig. S2C, lanes 3, 4, respectively). Only a slight reaction was observed with MGO-derived AGEs (Supplementary Fig. S2C, lane 2), however it was significantly weaker than that observed with MAGEs. Further, a specific anti-MAGE monoclonal antibody (Supplementary Fig. S3) designated MAGE/10 was generated in mice. This antibody showed specific and exclusive reactivity with MAGEs formed in a dry state (MWG) (Fig. 4A,B). Cross-reactivity was not observed with other products including those formed from conventional precursors like glc, fru, lac, GA or MGO. This finding agrees with similar observations using polyclonal anti-MAGE antibodies (Fig. 3D,E, Supplementary Fig. S2C and other not shown). We concluded that AGEs formed from mel constitute a distinct class of products differing from those referred in the literature as “glycation products”.

**Figure 5:**
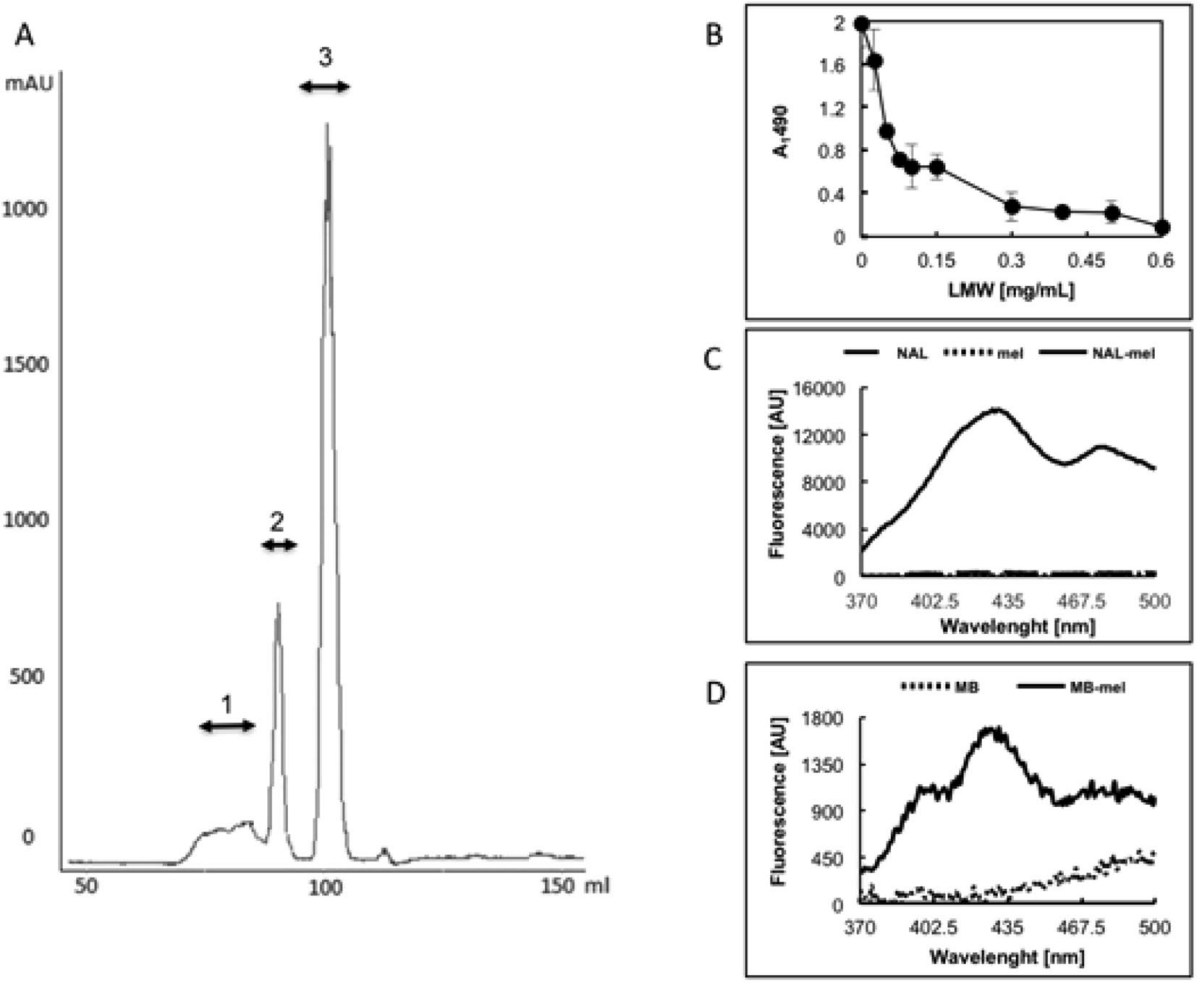
Purification and characterization of the LMW MAGE generated from mel and Nα-acetyl-lysine (NAL). (**A**) Elution profile of the LMW MAGEs generated on the HW-40S column recorded at 225 nm; the eluted material was pooled resulting in fractions 1, 2, and 3; (**B**) Fraction 2 was tested in competitive ELISA on a plate coated with 1 µg/well MB-mel; 0–600 μg/ml LMW MAGEs fr.2 was used as a competitor**;** fluorescence profile within 370–500 nm after excitation at 350 nm was recorded for 1 mg/ml solutions of (**C**) NAL, mel, LMW MAGEs and (**D**) protein-bound AGEs (MB, MB-mel).

**Figure 6:**
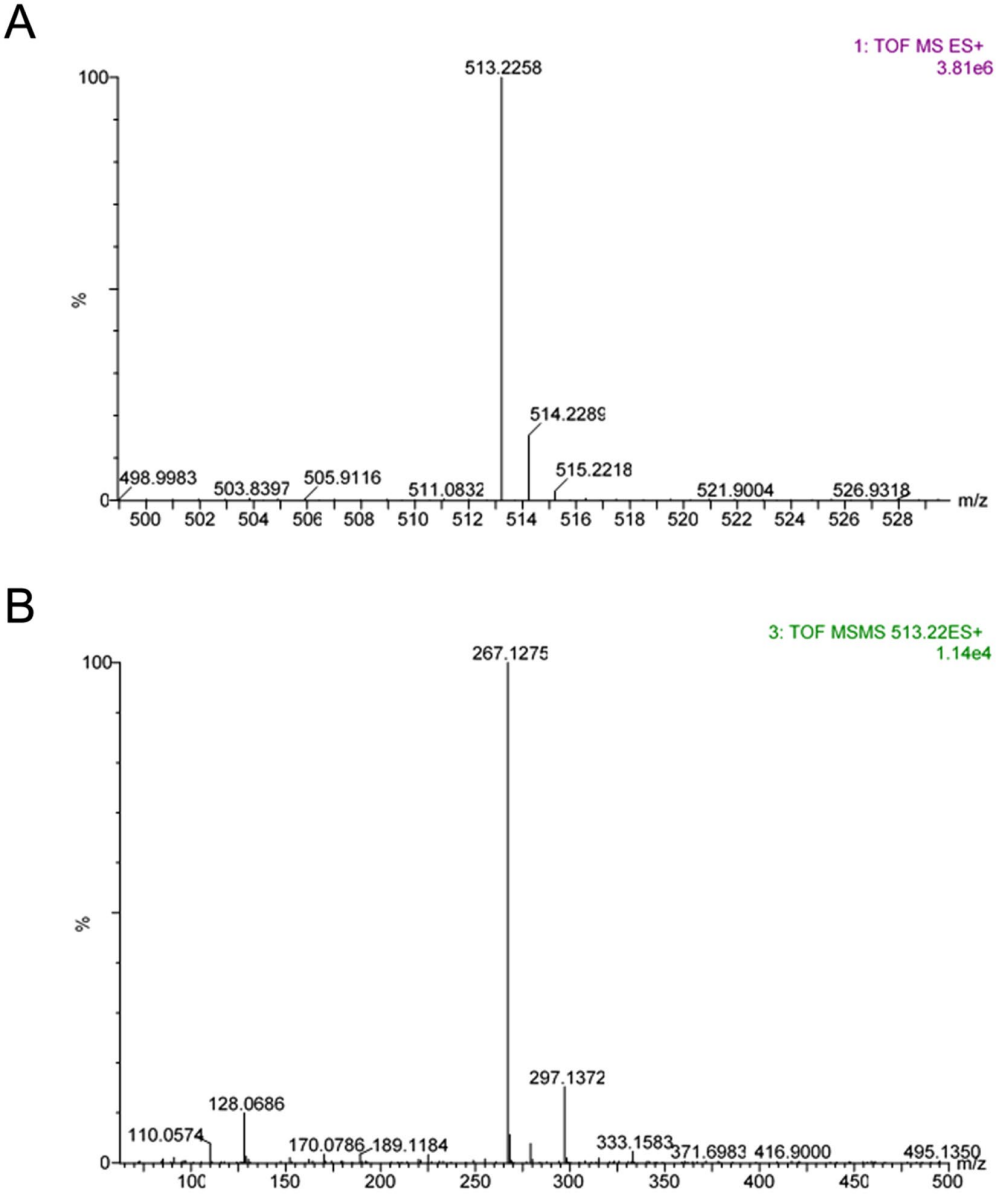
LC-TOF–MS analysis of the LMW MAGEs. (**A**) Mass spectrum of the major ion with *m/z* 513 and (**B**) its MS/MS fragmentation pattern.

In order to elucidate the structure of the specific epitope recognized by the anti-MAGE/10 mAb, we generated under MWG conditions the model low molecular mass adducts (LMW MAGEs) of N-α-acetyl-lysine (NAL) and mel. The purified product (described in “Methods”, Fig. 5A) was tested in a competitive ELISA with anti-MAGE/10 mAb (Fig. 5B) and was subjected to structural characterization. Fluorescent spectra (Fig. 5C) revealed two characteristic emission maxima at around 430 and 475 nm. Moreover, the spectrum resembled one of the MAGEs generated on MB (Fig. 5D). The predicted molecular mass of the LMW MAGEs generated from mel and NAL was 513 Da and was confirmed by LC-TOF–MS analysis (Fig. 6A). The daughter fragmentation ions derived from the major ion were of *m/z* 267 and 129 (Fig. 6B).

**Figure 7:**
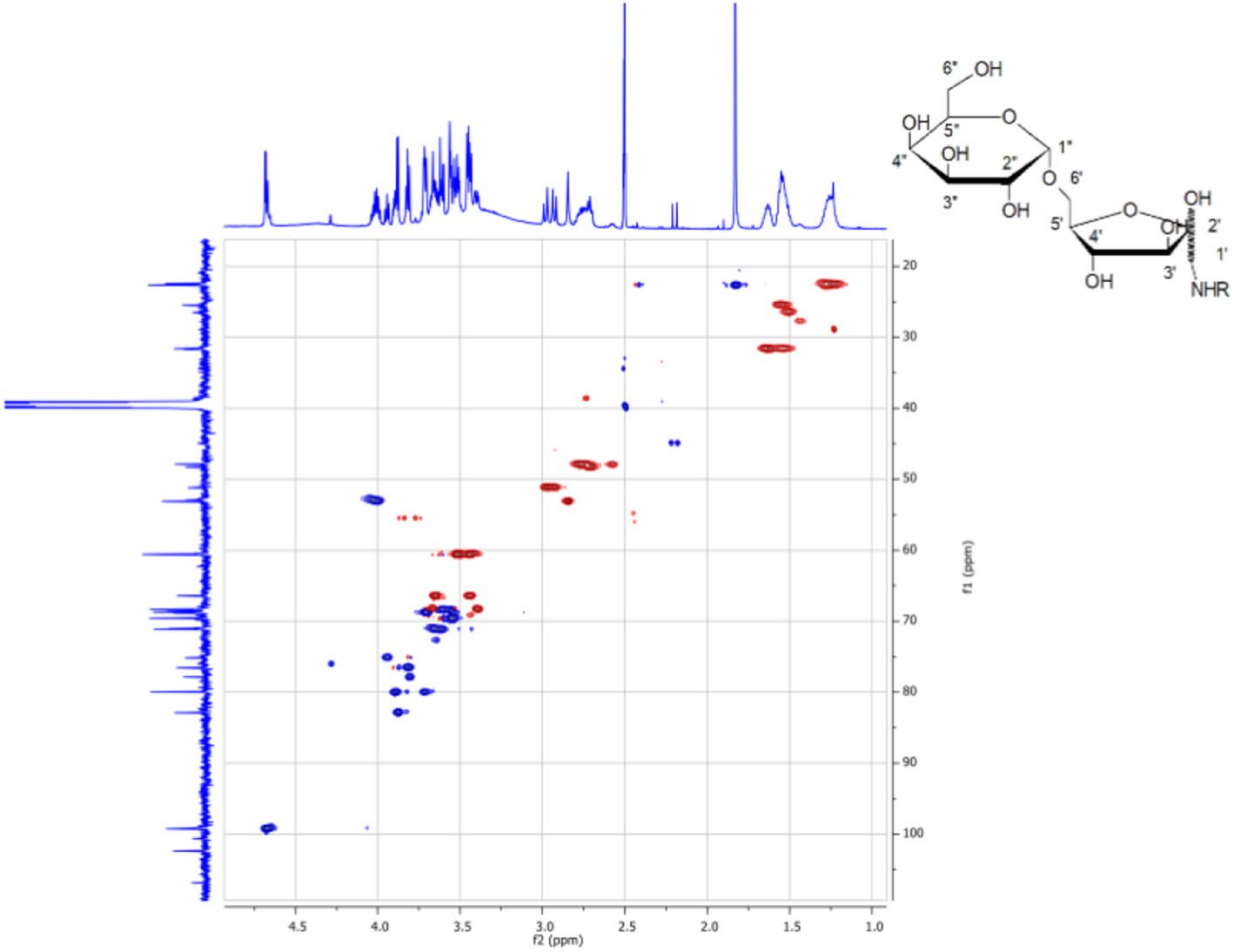
NMR spectrum of the LMW product formed from melibiose and *N*-α-acetyl-lysine. The inset shows the solved structure of the novel MAGE product.

The structure of LMW MAGEs was finally elucidated by a series of 1D and 2D NMR experiments (Fig. 7, Table 2) that confirmed the crosslink between the sugar and *N*-α-acetyl-lysine moiety. The complexity of 1D ^1^H NMR and ^13^C NMR was observed due to the presence of isomers of the fructosamine moiety, a product with open-chain and cyclic structures (Supplementary Fig. S4, product 1, 2 and 3, respectively). 2D NOESY confirmed the glycated site between H–N in *N*-α-acetyl-lysine and 1-α-C in the sugar. The multiple correlations in the fructose moiety of the ^1^H–^1^H COSY spectrum and in the ketoamine moiety of the ^1^H–^1^H COSY spectrum indicated the presence of isomers. We suggest that this was due to the mutarotation of the Amadori rearrangement product (Supplementary Fig. S4). The equilibrium mixture is about 59% β-isomer (compound **2**) and about 33% α-isomer (compound **3**), though there are traces, 8%, of the open chain form (compound **1**).

**Table 2:** The resonance assignments of ^1^H and ^13^C based on the NMR analysis of the novel MAGE.

Position	^1^H NMR δ (ppm)			^13^C NMR δ (ppm)		
	Isomer α	Isomer β	keto	Isomer α	Isomer β	keto
6	2.71	2.76	2.58	48.24	47.87	47.93
2-NH	7.70	7.64	7.77			
7				168.51	168.64	
8	1.8311	1.829	1.828	22.70		
1′	2.85	2.94; 2.97	2.58	53.120	51.22	48.00
2′				100.54	102.30	205.74
3′	3.90	3.88		80.05	82.94	
4′	3.82	3.80		76.57	77.81	
5′	3.72	3.95		80.04	75.18	
6′	3.67 3.403	3.63; 3.45		68.35	66.27	
1″	4.67 (br)	3.68 J = 3.7 Hz	4.65 J = 4.12 Hz	99.11	99.17	98.89
5″	3.08			70.98	71.07	70.87
6″	3.446 3.516			60.52	60.46	60.33

**Figure 8:**
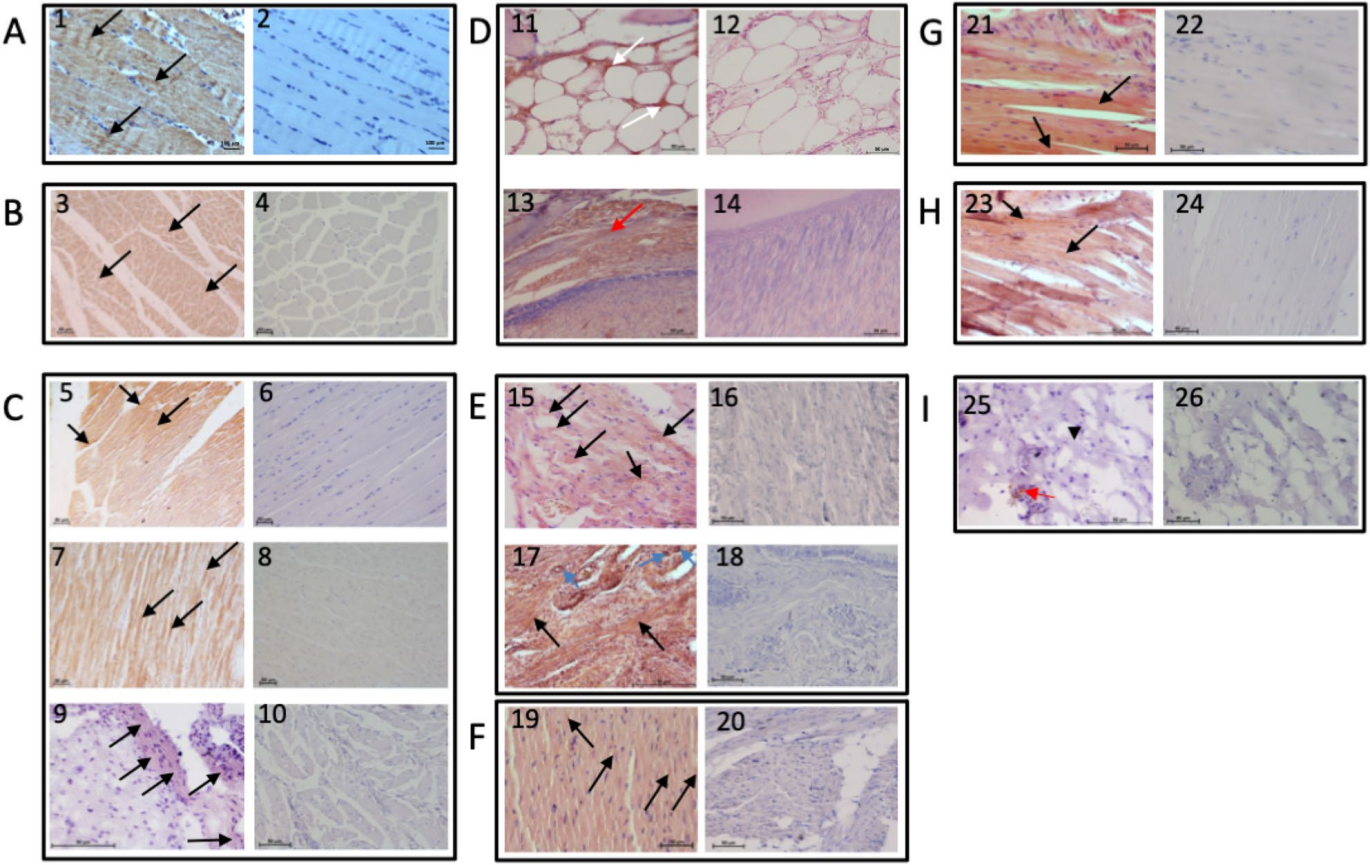
MAGE accumulation in human and animal muscles. Tissue sections from human (**A**), horse (**B**), pig (**C**), rabbit (**D**), rat (**E**), chicken (**F**), frog (**G**), fish (**H**), and snail (**I**) were stained with hematoxylin–eosin (H&E) in addition to the monoclonal antibodies anti-MAGE/10 (odd numbers) or with H&E only (even numbers). The representative pictures of human skeletal muscles (1, 2), horse skeletal muscles (3, 4), pig skeletal muscles (5, 6), pig heart (7, 8), pig intestinal smooth muscles (9, 10), rabbit adipose tissue (11, 12), rabbit connective tissue (13, 14), rat heart (15, 16), rat intestinal smooth muscles (17, 18), chicken heart (19, 20), frog skeletal muscles (21, 22), fish skeletal muscles (23, 24), snail muscle tissue (25, 26) are shown. Black arrows show positive staining of skeletal (1, 3, 5, 21, 23), heart (7, 15, 19) and smooth muscle (9, 17, 25) myocytes; black arrowhead shows negative staining of smooth muscle myocytes (25); blue arrows show positive staining of arterial myocytes (17); red arrows show positive staining of connective tissue (13, 25); white arrows show positive staining of adipose tissue (11). The scale bars show 50 μm.

Finally, the presence of MAGEs in human tissues as well as those from several animal species, namely horse, pig, rabbit, rat, chicken, frog, fish, and snail was assayed by immunohistochemistry using mAb anti-MAGE/10 as a probe. Staining revealed antibody binding to diverse tissues of the tested species. There was striking cytoplasmic reactivity with skeletal (Fig. 8 panel 1, 3, 5, 21, 23) and cardiac myocytes (panel 7, 15, 19) of all tested species. The chicken heart displayed the most uniform, intense staining (panel 19), while smooth muscle myocytes from different animals showed the most variable staining ranging from very intense in rat (Fig. 8E, panel 17—black arrows show intestine and blue arrows show arterial muscles) to minimal in pig (Fig. 8C, panel 5, black arrow—intestinal muscles) and no reactivity in snail (Fig. 8I, panel 25—arrowhead). We also appreciated intense staining of connective tissue from rabbit and snail (respectively panel 13, 25—red arrows) as well as adipose tissue from rabbit (Fig. 8D, panel 11—white arrow). Immunohistochemical analysis of human skeletal muscle with polyclonal anti-MAGE antibody revealed that there is more intense staining of diabetic patient muscle than physiological pattern of healthy person (Supplementary Fig. S5).

## Discussion

In this study we report on the identification of a novel AGE (MAGE) that is an analog of a model adduct formed in vitro in a dry state (MWG) during reaction of melibiose with protein. Structural properties of the novel AGEs obtained in vitro from mel are distinct from the conventionally studied adducts derived from other sugars like glc, rib or fru. The in vivo counterpart of a model MAGE seems to be more immunogenic in human (inducing autoantibodies) in comparison to AGEs formed from other carbohydrates. We have established that MAGE has unique immunochemical properties using the generated monoclonal antibodies. The structure of the MAGE has been resolved by mass spectrometry and NMR analysis, showing a mixture of isomers containing a fructosamine moiety with open-chain and cyclic structures. In contrast to fructosamine formed from glc or fru, MAGE contains an attached disaccharide where both carbohydrate moieties (i.e. gal and glc) are in a closed form^35^. Interestingly, the products formed from mel using aqueous conventional glycation (ACG) showed different antigenic properties when compared to MWG MAGEs (Fig. 4), suggesting that the dry state favors unique rearrangements allowing retention of both an intact and closed form of the attached carbohydrate. A structural analog of this model adduct obtained in vitro appears to be generated in vivo and accumulates in animal and human tissues (Fig. 8, Supplementary Fig. S5). The origin of this natural MAGE remains to be elucidated along with its detailed structure and biological role. We observed that serum from diabetic patients reacted with our model MAGE (Fig. 1B,D) suggesting that, like other AGEs^32,33^, this product may induce autoimmunity. In contrast, unsuccessful reactivity of human serum with AGEs derived from glc under HTG suggests that these in vitro formed products do not have a counterpart autoantibodies. These data show that hyperglycemia may induce preferential in vivo generation of MAGEs (or some structural mimetops) resulting in an immune reaction. This process may require chronic antigen exposure, as it took several immunizations with a mixture of different protein-MAGEs to generate anti-MAGE antibodies in mice (see “Methods”). The other authors have observed similar types of cross reactivity^36–38^. The in vivo biosynthetic pathway of native MAGE formation and whether MAGEs can be delivered with diet remain to be elucidated. Since melibiose is generated during food fermentation by *Bifidobacterium*^27,39^, *Lactobacillus*^40^, *Lactococcus*, *Leuconostoc* sp. and yeasts^41^, one can hypothesize that this process may provide the substrate source for further MAGE generation. Since there are no data available on intestinal MAGE formation and subsequent absorption into the circulation, it remains to be seen whether exogenous melibiose is absorbed in the intestines. It will be also worth studying whether in galactosemia there is presence of adducts structurally similar to MAGEs. This might be supported by our data showing reactivity of some autoantibodies present in human serum with MB-gal (Fig. 1B, lane 7).

Finally, the generated mAb anti-MAGE will be useful as a tool for studying the biological role of this novel AGE. Anti-MAGE immunostaining showed common cytoplasmic accumulation in metabolically active tissues, such as muscle, although collagen or extracellular matrix with lower protein turnover were also recognized (Fig. 8). In mollusks, specific reactivity was observed in stromal cells rather than myocytes. Whether MAGE is a marker of enhanced/abnormal metabolism will be the subject of further study. Additional tissues, including blood, kidney, brain, and skin will also be screened in the future for the presence of MAGE and its association with various pathologies, such as cancer.

It should be noted that adduct formed in vitro in a dry state (MWG) during reaction of melibiose with protein is distinct from the products formed in water solution (ACG). Water molecules as integral element on protein surface form solvating layer even in dried state after lyophilization where pure liquid water is absent. The first solvating layer is an ordered structure formed with hydrogen bonds of water and hold with protein molecule, facilitating interaction with ligand^42^. It mimics cellular environment, with the solvating layer on proteins and formation of distinct product.

In conclusions, we have identified the novel structure of unique AGE (MAGE) that can be formed in vitro from melibiose. NMR data revealed that protein glycation by melibiose results in a mixture of isomers with open-chain and cyclic form of the fructosamine moiety. The naturally present structural analog of MAGE was found to accumulate in tissues of different animal taxonomic classes within both invertebrate and vertebrate up to human. Generation of the specific anti-MAGE antibody allows for studying MAGE’s role in biology. This novel glycation adduct will also spin out further proteomic studies on protein modifications opening new field of research.

## Methods

### Materials

All chemicals were from Sigma-Aldrich (Saint Louis, MO, USA), unless otherwise stated. The proteins used for glycation, i.e. myoglobin from equine skeletal muscle (MB), lysozyme (LYS), and rabbit gamma globulin (RIg) were used in form as purchased and bovine serum albumin (BSA) was first purified on gel filtration column (HW-55S Toyopearl resin, Tosoh-Bioscience; XK16/100 column, Pharmacia, Sweden) as described^43^. The obtained monomer fraction was dialyzed against water and lyophilized before glycation. LC/MS-grade water, acetonitrile, and formic acid were purchased from J.T. Baker (Deventer, Netherlands). A Leucine Enkephalin was purchased from Waters (Milford, USA). We used the following secondary antibodies: goat anti-rabbit IgG-horseradish peroxidase (HRP), anti-rabbit IgG-alkaline phosphatase, goat anti-mouse immunoglobulins-HRP (DAKO, Glostrup, Denmark) and goat anti-human IgG-HRP (ICN Biomedicals, Irvine, CA), goat anti-mouse IgE-HRP (Acris, Herford, Germany), horse anti-mouse Igs-HRP. All methods were carried out in accordance with relevant guidelines and regulations.

### Synthesis of model protein AGEs

The protein AGEs were obtained in different glycation conditions: dry state high-temperature glycation (**HTG**), microwave glycation in dry state (**MWG**), high-pressure glycation in aqueous solution (**HPG**) and aqueous conventional glycation (**ACG**). In order to generate glycation products we prepared a miliQ water solution mixtures of protein monomer and the respective carbohydrate: glucose (glc; Serva, Heidelberg, Germany), galactose (gal), ribose (rib, Loba-Chemie; Mumbai, India), fructose (fru; Merck, Darmstadt, Germany), mannose (man), lactose (lac), cellobiose (cel, Merck, Darmstadt, Germany) melibiose (mel; Fluka, St. Louis, MO, USA), maltose (mal; Merck/Bioshop, Burlington, ON, Canada) or dicarbonyl—methylglyoxal (MGO, distillate), glycolaldehyde (GA) or lipid derivative—acrolein (ACR), trans-2-nonenal (T_2_N, Steinheim, Germany) and 4-hydroxynonenal (HNE, Billerica, USA). The solutions for HTG were lyophilized and incubated in the oven at 120 °C for 120 min or subjected to the microwave set up at 85 °C for 45 min for carbohydrates and at 60 °C for 15 min for aldehydes in order to obtain MWG products. In comparison the mixture of substrates dissolved in PBS was incubated at 37 °C for 21 days to generate ACG products or was subjected to high pressure to obtain HPG products as described earlier^21^. The products formed on proteins were next fractionated based on extent of crosslinking and molecular mass. The gel filtration method on Sephadex G-200 (Pharmacia, Sweden) XK16/100 column in PBS was used for this purpose. The material present in the individual fractions was pooled, dialyzed against water and lyophilized for further characterization.

### Synthesis of low molecular mass (LMW) AGE

The 1:1 mixture of *N*-α-acetyl-lysine and melibiose dissolved in the miliQ water was lyophilized and subjected to MWG in a microwave reactor (Initiator, Biotage, Uppsala, Sweden), equipped with a pressure compressor (Jun-Air Model 2xOF302-40B 230 V/50 Hz) set up at the 200 W (60 °C) for 25 min. The resulting products were next dissolved in 0.1 M ammonium acetate buffer pH 6.8 and subjected to the ultra-wave water bath (Bandelin Sonorex) for 15 min in order to solubilize the pellet. The undissolved products were separated by centrifugation at 10,000×*g* for 15 min. The generated AGEs were separated on HW-40S column (1.6 × 100 cm, Toyopearl, Tosoh) in the 0.1 M acetate buffer, pH 6.8 using the FPLC system (ÄKTA Explorer, Amersham Biotech, Sweden). The eluent was monitored at 225, 297 and 325 nm and the obtained fractions were tested with the anti-MAGE Ab. The reactive material was pooled, desalted on the Bio-Gel P2 column (Bio-Rad, Richmond, CA, USA) in the miliQ water, and lyophilized.

### Carbohydrate content analysis using gas–liquid chromatography coupled with mass spectrometry (GLC-MS)

The MAGEs formed on MB were analyzed for the carbohydrate content after acid hydrolysis and identification of the alditol acetates, as published earlier^21^.

### NanoUPLC-Q-TOF-ESI–MS/MS analysis

The samples were analyzed using nanoAcquity Ultra Performance LC system combined with Xevo G2-Q-TOF mass spectrometer (Waters, Milford, USA). The LC system was equipped with HSS C18 analytical column (50 mm × 1 mm; 1.8 µm) purchased from Waters (Milford, USA). The sample was fractionated at a flow-rate of 30 µL/min, at 35 °C using the gradient of the mobile phase A (0.1% of formic acid in water) and B (0.1% of formic acid in acetonitrile) with the following steps: (1) initial condition—5% B; (2) 0 to 1 min—5 to 30% B; (3) 1 to 2 min—60% B; (4) 2.10 min—5% B. The weak and strong wash solvents were 5% acetonitrile in water and acetonitrile, respectively. The mass spectrometer was equipped with an electrospray ionization source. The instrument was run at the capillary and cone voltage of 3.0 kV and 40 V, respectively. The desolvation gas flow was set at 500 L/h and constant temp of 450 °C. The source temperature was set at 100 °C and the cone gas flow rate was 20 L/h. Spectra for positive charge ions were acquired in sensitivity mode from *m/z* 80 to *m/z* 600. The Leucine Enkephalin was used as a lock mass solution in the accurate mass measurement.

### NMR analysis

The freeze-dried material was dissolved in dimethyl sulfoxide-D6 (DMSO-6, 500 µL) and transferred to a 5 mm NMR tube for NMR analysis. All NMR experiments were performed on a Varian spectroscope equipped with a 5 mm TBO probe and operated at 25 °C (298 K) with a proton frequency of 598 MHz. The chemical shifts (δ values), given in parts per million (ppm), were referenced to the signals of the residual protons (2.50 ppm) and carbon atom (C39.5 ppm) in DMSO-6. All 1D (^1^H, ^13^C, and DEPT-135) and 2D (^1^H–^1^H COSY, ^13^C–^1^H COSY, and NOESY) NMR measurements were performed using standard Varian pulse sequences. Sweep widths of 5000 and 25,000 Hz were used in ^1^H and ^13^C NMR, respectively. 2D ^1^H–^1^H COSY and ^13^C–^1^H COSY spectra were collected in quadrature with 1024 points in 2 and 256 points in 1, and the sweep widths were 5000 and 15,000 Hz of ^1^H and ^13^C dimensions, respectively. 2D NOESY was recorded with mixing time of 400 ms and 2561 increments containing 16 transients of 2048 complex data points. 2D NMR data were applied with a 90° phase-shifted, squared sine-bell window function in both dimensions prior to Fourier transformation. Data were interpreted as described^44^.

### Generation of polyclonal anti-MAGE antibodies

Rabbits were injected intradermally with a mixture of MB-mel fr. 1 and fr. 2 dissolved in PBS with addition of the complete Freund adjuvant. The generated anti-MAGE antibodies present in the rabbit serum were affinity purified on the set of columns with Sepharose-immobilized MB-mel, MB or on agarose-melibiose. In order to make the affinity columns 16 ml of Sepharose gel (GE Medical Systems Polska, Warsaw, Poland) was first activated with 2.2 g of CNBr for 25 min at room temp and constantly maintained pH 11. The activated gel was washed with 0.1 M NaHCO_3_, pH 8.2 and either 30 mg in 5 ml of MB or 15 mg in 3.5 ml of MB-mel fr.1 and fr.2 mixture in 0.1 M NaHCO_3_ was added. The coupling reaction was carried for 2 h at room temp and the free amine groups were blocked with 1 M ethanolamine for 2 h at room temp following overnight incubation at 4 °C. Next day after washing with water and 2 h incubation with 2 M K_2_HPO_4_ the gel was packed into the glass column and equilibrated with PBS before antibody purification. The diluted in PBS serum was treated with ammonium sulfate to precipitate antibodies and centrifuged. The obtained pellet dialyzed against PBS was concentrated and loaded on the column with Sepharose-MB-mel. The bound antibodies eluted with 3 M KSCN were dialyzed and further fractionated on the next column with Sepharose-MB in order to remove the Ab binding carrier protein. The unbound material was collected and subjected to the final column filled with Agarose-melibiose (Sigma) to separate out the antibody binding melibiose. The purification procedure of the specific anti-mel-AGE Abs is shown in “Results” section on Supplementary Fig. S1.

### Generation of monoclonal antibody binding MAGEs

All animal experiments were approved by the Local Animal Care and Use Committee at the Hirszfeld Institute of Immunology and Experimental Therapy PAS (LKE 53/2009). The Balb/c mice were injected with 1 mg/ml each of the MB-mel and RIg-mel. After a month, the serum was tested in ELISA (described below) for reactivity with different carrier proteins, namely MB, BSA and RIg glycated with melibiose. The injections were repeated 6 times every 2 weeks to modulate high response against the MAGEs and low against the carrier proteins. The last booster injection was performed with the MB-mel in order to diminish induction of the anti-RIg antibodies. Next, the hybridomas producing anti-AGE Abs were generated by fusion of the spleen cells from the mel-AGE-injected mouse with the SP-2/0 myeloma cells (ATCC) and selected with the medium containing HAT (hypoxantine/aminopterin/thymidine). In order to identify the clones producing specific antibodies the ELISA on plates coated with MB-mel, BSA-mel and RIg-mel or Lys-mel was performed. The control wells were coated with the respective carrier proteins. The reaction was visualized using the secondary antibodies binding all mouse immunoglobulin classes conjugated with the HRP. The cells producing most abundant amount of the specific antibodies were subcloned to isolate single clones that were next propagated for antibody production. The individual clones were subjected to isotyping with the Rapid Mouse Isotyping Kit—Gold Series, LFM-ISO-1-5 (RayBiotech Inc., Norcross, GA). For further experiments we have utilized the clone number 10 (anti-MAGE/10) producing mAb classified as mouse IgE.

### SDS-PAGE and Western blotting analysis

SDS-PAGE was performed as described earlier^21^. Briefly, glycated or unmodified proteins were loaded on gel in reducing conditions and either stained with Coomassie Brilliant Blue (CBB) or transferred onto Immobilon P (0.45 μm; Millipore, USA). Membrane was incubated for 2 h, at room temp with one of primary antibody: human serum (diluted 50 times), rabbit serum (diluted 50 times), anti-MAGE affinity-purified rabbit antibody (diluted 500 times) or cell culture supernatant (undiluted) from clones producing mouse antibody. The respective secondary antibody anti-human IgG-HRP (1:20,000), anti-mouse immunoglobulins-HRP (diluted 1:2,000) or anti-rabbit IgG-AP (diluted 1:20,000) were used. The reaction was detected with ODP or AP substrates, according to standard procedure.

### ELISA and competitive ELISA

Microplate was coated with 0.5–1 μg/well of MB-mel (purified fr.2, containing cross-linked products), MB-mal, MB-cel or MB-lac dissolved in 100 μl of carbonate buffer pH 9.6 at room temp for 4 h. As the control, some wells were coated with the respective carrier protein, i.e. unmodified MB, BSA or RIg. After 3 washing cycles with the TBS-T washing buffer (20 mM Tris–HCL, 50 mM NaCl/0.05% Tween-20, pH 7.4) the plate was blocked with 4% non-fat dry milk (NFDM)/TBS-T at 4 °C, overnight. Following 3 times washing with TBS-T the wells were incubated for 2 h with 100 μl of the cell culture supernatant containing anti-MAGE/10 mAb or purified polyclonal anti-MAGE Abs diluted in PBS 2500 times or 8× (dilution of hybridoma cells supernatant), respectively. For the competitive ELISA, the cell culture supernatant containing anti-MAGE/10 mAb was pre-incubated with LMW MAGE (0–600 μg/ml) at room temp for 1.5 h before applying to the microplate. Following washing with TBS-T (3 times), 100 μl of the respective secondary antibody coupled with HRP—anti-mouse immunoglobulins or anti-rabbit IgG diluted 2000 times in PBS were applied for 1 h, at room temp. Next, 3 times washing with TBS-T was performed and the reaction of the Ab-bound HRP was developed using 50 μl/well of the ODP (*O*-phenylenediamine dihydrochloride) substrate and stopped with 50 μl/well of 40% H_2_SO_4_. The absorbance at λ490 nm was recorded and data presented as A_1_490 after normalization by subtraction of the absorption generated by secondary Ab (PBS in place of serum). In the experiments with sera from diabetic (DM), diabetic nephropathy (NPH) and Buerger’s disease patients, the samples were diluted 4 times in PBS and as the secondary Ab we used goat anti-human IgG-HRP diluted 20,000 times.

### Immunohistochemistry

The human tissue was collected at the tissue bank of the Department of Pathology at Wroclaw Medical University after the approval from the Bioethics Committee of the Medical University in Wroclaw. The samples were fixed in 4% buffered paraformaldehyde (PFA)/PBS and embedded in paraffin (FFPE) for cutting into 4 μm sections. The slices mounted on poly-l-lysine coated glass slides were first deparaffinized by heating at 60 °C and then immersed in xylene for 9 min. The sections were immunostained utilizing the ABC DAKO kit. The endogenous peroxidase was first blocked with the blocking reagent and the sections were placed in distilled water at room temperature (15 min) followed by antigen retrieval in citric acid buffer pH 6.0 (2 × 8 min, heating in microwave Daewoo at 350 W and leaving at room temperature). The 30 min incubation at room temp was performed with swine serum diluted 1:50 in Tris-buffered saline solution (TBS) containing Tween 20 (20 mM Tris–HCL, 50 mM NaCl, 0.05% Tween-20, pH 7.5) as blocking step. After washing with distilled water the sections were incubated with the primary anti-MAGE antibodies (affinity-purified rabbit IgG diluted 1:100 in PBS or mouse anti-MAGE/10 undiluted cell culture supernatant) for 1 h, at room temp. The washed with TBS sections were next subjected to the 30 min incubation with LSAB reagent or anti-mouse IgE secondary antibody (HRP, diluted 1:500) and after another TBS washing the reaction was developed with 3,3′-diaminobenzidine tetrahydrochloride (DAB) for 5 min. Finally, the slides were counterstained with hematoxylin–eosin (HE) and mounted under coverslips. The slides were observed under the Olympus BX51 microscope and pictures were recorded with the camera.

In case of animals the tissue samples were obtained from the Department of Animal Physiology and Biostructure at Wroclaw University of Environmental and Life Sciences. The animals, from which the tissues were derived, were bred in vivariums, pens and stables at the University of Environmental and Life Sciences in Wroclaw. Tissue samples were collected during other experiments conducted at the university. Animal tissue samples were stained as described for the human tissue with some modifications. The 7 μm sections were subjected to staining with ImmPRESS Universal Reagent Kit (Vector Laboratories, Burlingame, CA, USA). The endogenous peroxidase was neutralized using 0.03% H_2_O_2_ for 25 min on the deparaffinized tissue sections. After blocking with 2.5% normal horse serum for 30 min the mouse anti-MAGE/10 undiluted cell culture supernatant was applied and incubated at room temp for 1 h. After washing with PBS, the HRP-conjugated anti-mouse IgE secondary antibody was applied for 30 min and the peroxidase activity was developed. The samples were next counterstained with Mayer's hematoxylin, dehydrated and mounted in Pertex (HistoLab, Göteborg, Sweden).

### The approval and accordance statements

The study was carried out in compliance with the ARRIVE guidelines.

## Supplementary Information

Supplementary Information.
