# Supplementary material for: The melibiose-derived glycation product mimics a unique epitope present in human and animal tissues
Source: Sci Rep. 2021 Feb 3;11:2940. doi: 10.1038/s41598-021-82585-7 (PMC7859244; doi:10.1038/s41598-021-82585-7)

The melibiose-derived glycation product mimics a unique epitope present in human and animal tissues

Magdalena Staniszewska[1,2], Agnieszka Bronowicka-Szydełko[3], Kinga Gostomska-Pampuch[1,3], Jerzy Szkudlarek[1], Arkadiusz Bartyś[1], Tadeusz Bieg[4], Elżbieta Gamian[5], Agata Kochman[6], Bolesław Picur[7], Jadwiga Pietkiewicz[3], Piotr Kuropka[8], Wiesław Szeja[4], Jerzy Wiśniewski[3], Piotr Ziółkowski[5], Andrzej Gamian[1,3,9]*

[1]Hirszfeld Institute of Immunology and Experimental Therapy, Polish Academy of Sciences, Weigla 12, 53-114 Wroclaw, Poland, [2]Centre for Interdisciplinary Research, The John Paul II Catholic University of Lublin, Konstantynow 1J, 20-708 Lublin, Poland, [3]Department of Medical Biochemistry, Wroclaw Medical University, Chalubinskiego 10, 50-368 Wroclaw, Poland, [4]Department of Organic Chemistry, Bioorganic Chemistry and Biotechnology, Silesian University of Technology, Krzywoustego 4, 44-100 Gliwice, Poland, [5]Department of Pathomorphology, Wroclaw Medical University, Marcinkowskiego 1, 50-368 Wroclaw, Poland, [6]Department of Pathology, University Hospital Monklands, Monkscourt Ave, Airdrie ML6 0JS UK, [7]Faculty of Chemistry, University of Wrocław, 50-383 Wrocław, Poland, [8]Department of Anatomy and Histology, Wroclaw University of Environmental and Life Sciences, Norwida 1, 50-375 Wroclaw, Poland, [9]Wroclaw Research Centre EIT+, PORT, Stabłowicka 147/149, 54-066 Wrocław, Poland

* Correspondence and requests for materials should be addressed to A.G. (email: andrzej.gamian@hirszfeld.pl

Supplementary Figure legends

Fig. S1. Purification of the anti-MAGE polyclonal antibodies from rabbit antiserum.

Fig. S2. Characterization of the affinity-purified anti-MAGE polyclonal Ab
A) Testing of the affinity-purified rabbit Abs in WB and ELISA; the samples of 1. MB-mel and 2. MB were loaded on 12.5 % SDS-PAGE gel (with indicated molecular markers) and stained with Coomassie stain (**a1)** or transferred onto PVDF membrane for Western blotting with the affinity-purified polyclonal anti-MAGE antibodies (**a2**). Panel **a3** shows ELISA on a plate coated with MB-mel and reactivity with the affinity-purified antibodies. The data expressed as A_1490 (normalized by subtracting the absorbance for the well without primary Ab) are the mean of 3 wells and were normalized by subtracting the absorbance for the wells without primary Ab. **B)** Cross-reactivity of affinity-purified rabbit anti-MAGE Abs with the MAGEs formed on BSA as the carrier protein. SDS-PAGE on 8% gel **(b1)** and immunoblotting **(b2)** of BSA-mel separated according to molecular mass by gel filtration into **1**-**4** as fractions 1-4, respectively and **5.** BSA. C) Reactivity of affinity-purified rabbit anti-MAGE Abs with selected from different experiments AGE obtained under HPG (lines 1-4) or HTG (5) conditions; 1. MB-mel fr.2, 2. MB-MGO fr.1, 3. MB-fru fr.2, 4. MB-lac fr.2, 5. MB-mel fr.2, 6. MB.

Fig. S3. The reactivity of the anti-MAGE monoclonal antibody
The samples of MB-mel (lane 2) and molecular mass marker (lane 1) were loaded on 12 % SDS-PAGE gel and stained with Coomassie Brilliant Blue (**A)** or transferred onto PVDF membrane for Western blotting with the mAb anti-MAGE/10 **(B)**. The reactivity of antibody on an ELISA plate coated with 0.5 µg/well of MB-mel and MB is shown on Panel **(C).** The results are expressed as A_1490 and were normalized by subtracting the absorbance for the well without primary Ab.

Fig. S4. Proposed pathway of the MWG reaction of melibiose with Nα-acetyl lysine leading to fructoselysine, open-chain intermediate and isomeric forms of the novel MAGE

Fig. S5. Immunohistochemical pattern of skeletal muscle tissue from diabetes mellitus patient (A) and healthy person (B) stained with affinity-purified anti-MAGE polyclonal Ab. Negative staining control is shown on Fig. 8.2.

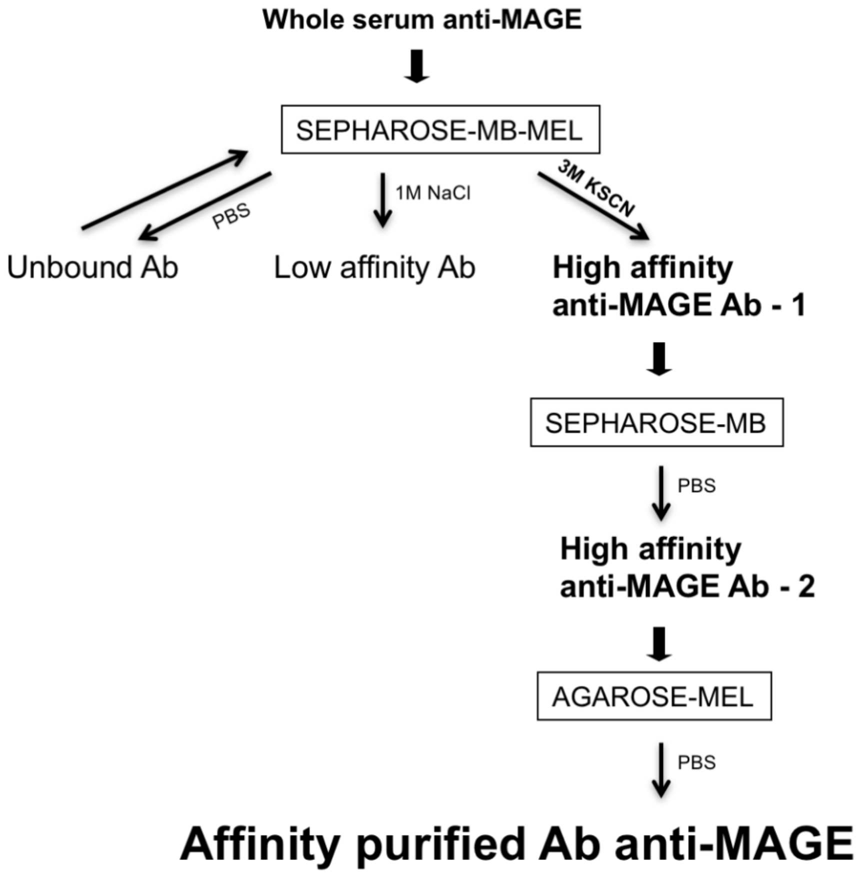
Whole serum anti-MAGE
SEPHAROSE-MB-MEL
PBS
1M NaCl
3M KSCN
Unbound Ab
Low affinity Ab
High affinity anti-MAGE Ab - 1
SEPHAROSE-MB
PBS
High affinity anti-MAGE Ab - 2
AGAROSE-MEL
PBS
Affinity purified Ab anti-MAGE

Fig. S1.

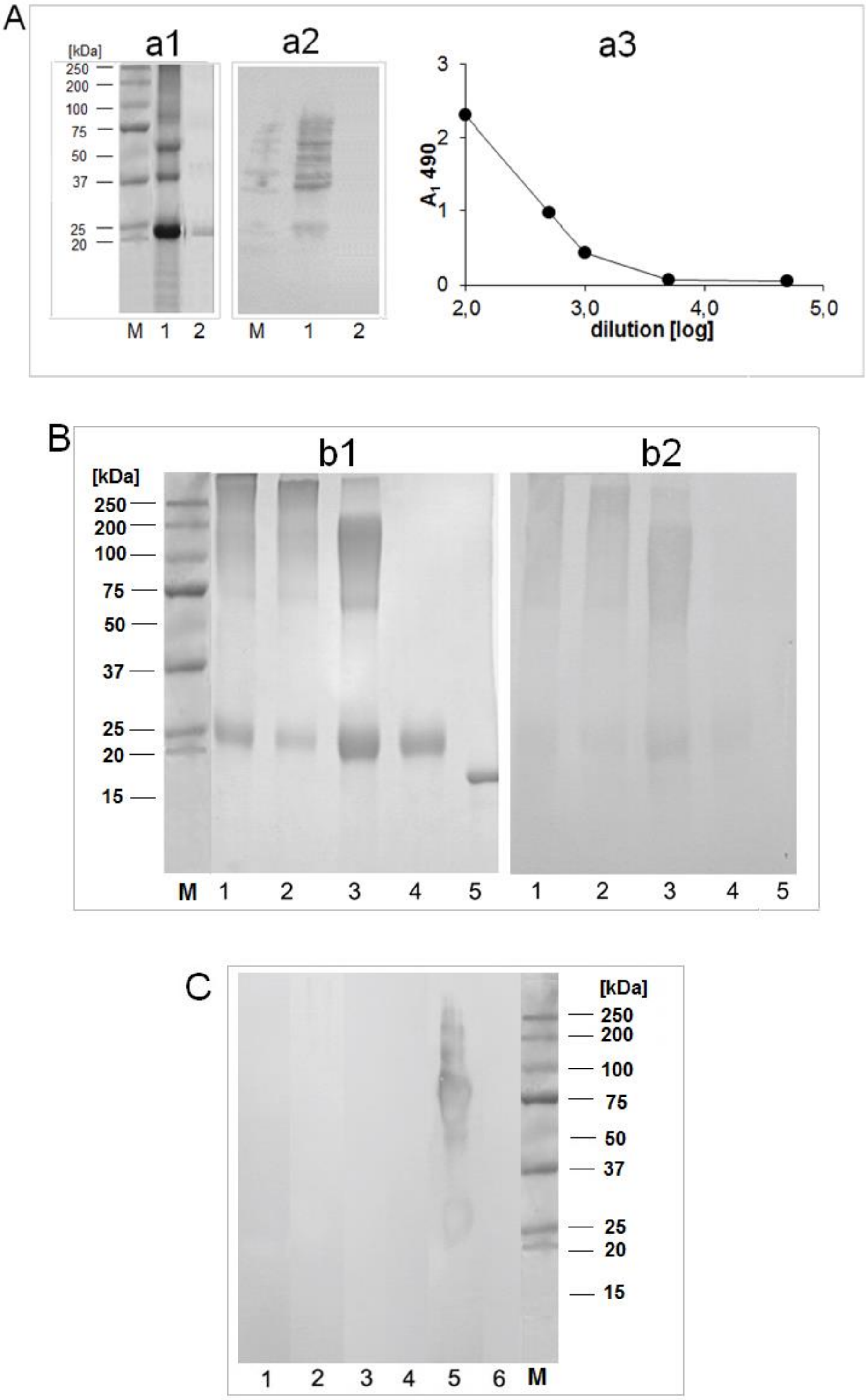
A
a1
[kDa]
250
200
100
75
50
37
25
20
M 1 2
a2
M 1 2
a3
3
2
1
0
A_1 490
2,0
3,0
4,0
5,0
dilution [log]
B
b1
[kDa]
250
200
100
75
50
37
25
20
15
M 1 2 3 4 5
b2
1 2 3 4 5
C
[kDa]
250
200
100
75
50
37
25
20
15
1 2 3 4 5 6 M

Fig. S2

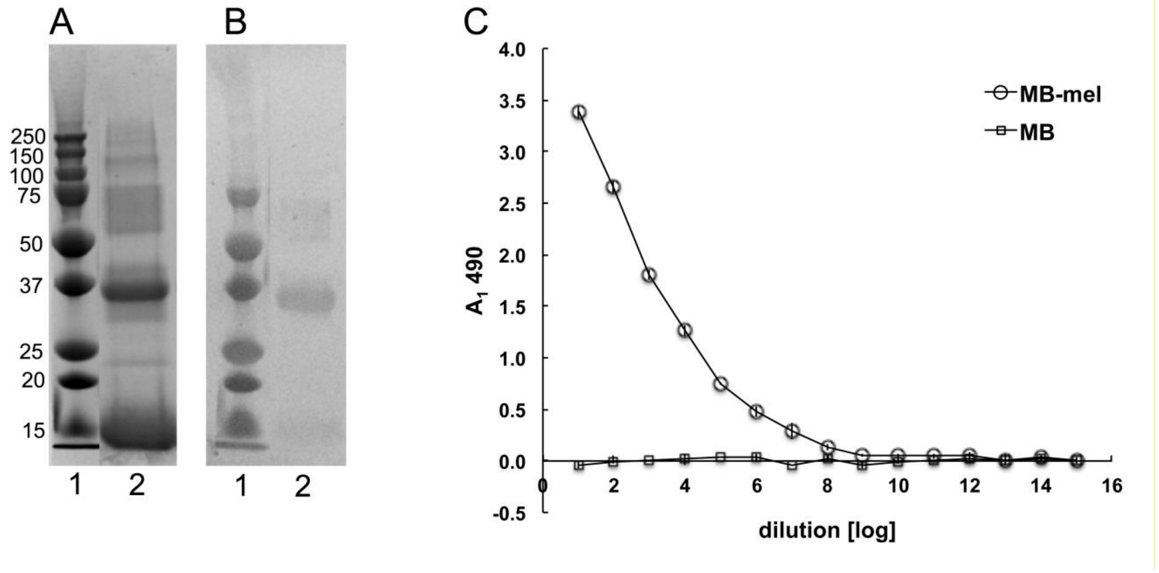
A
B
C
250
150
100
75
50
37
25
20
15
1
2
1
2
4.0
3.5
3.0
2.5
2.0
1.5
1.0
0.5
0.0
-0.5
A1 490
0
2
4
6
8
10
12
14
16
dilution [log]
MB-mel
MB

Fig. S3

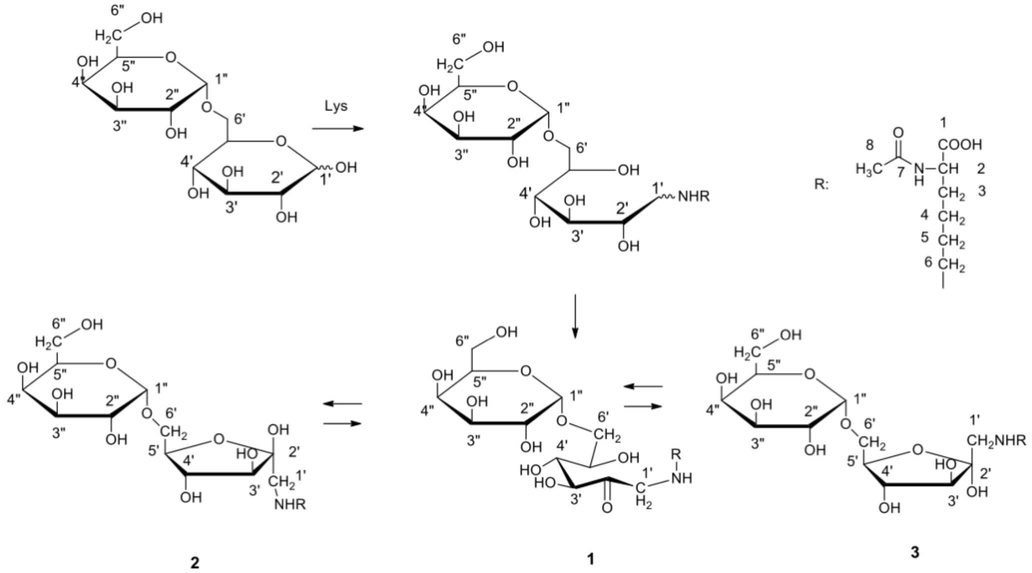

Fig. S4

A B

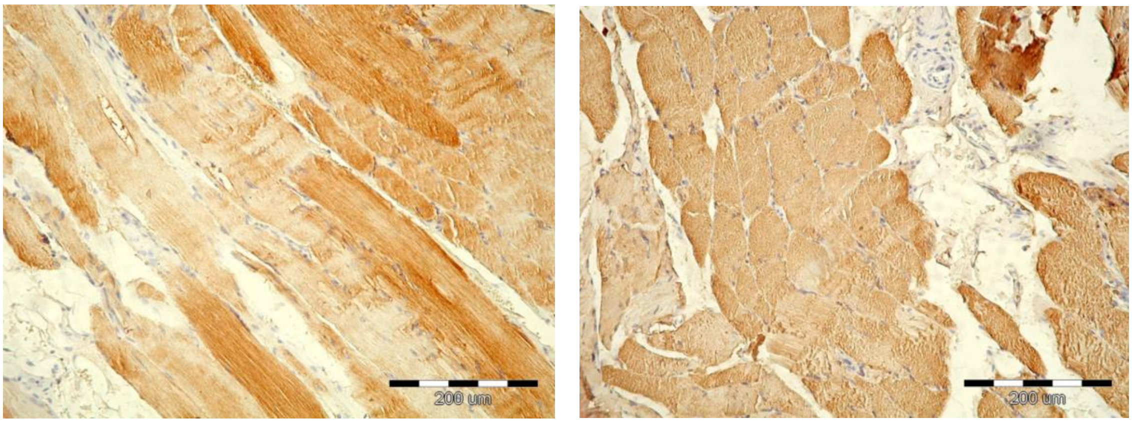

Fig. S5

Supplementary Information

Fig. S6. The full-length gel (A) and blot (B) of the cropped parts in the Fig. 1. The numbers indicate samples shown as in the main Fig. 1A and B, respectively.

Fig. S6

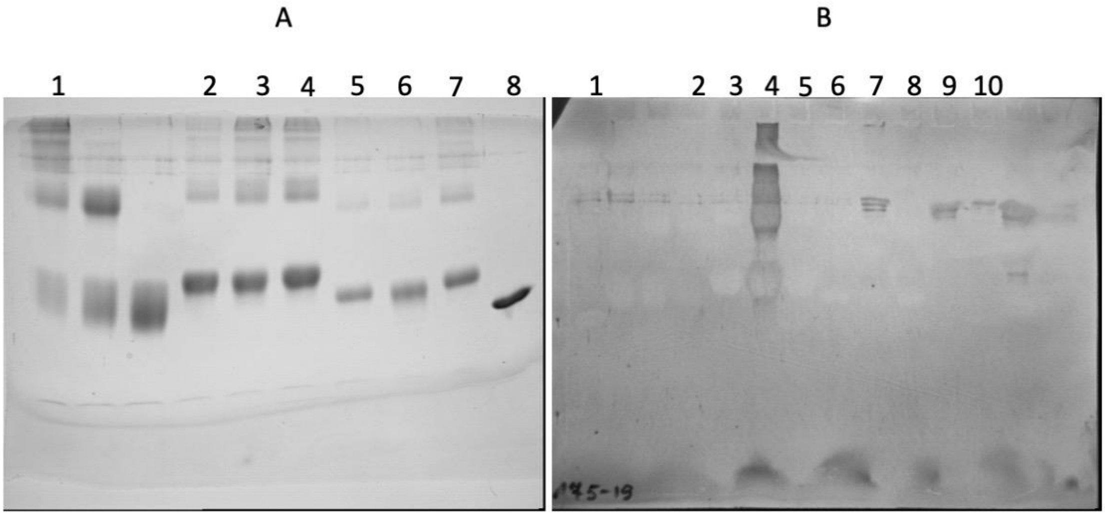

Fig. S7. The full-length gel of the cropped parts in the Fig. 2. The numbers indicate samples shown as in the main Fig. 1B.

Fig. S7

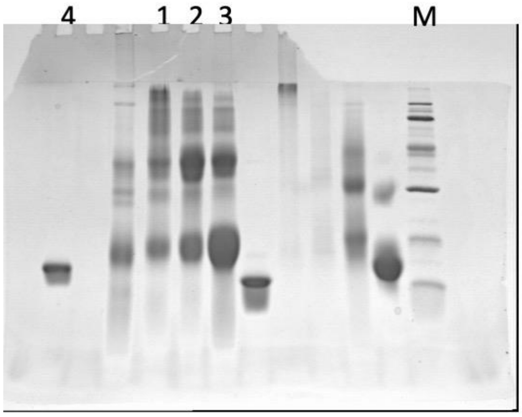

Fig. S8. Original gels and blots of the cropped parts presented in the Fig. 3. The numbers indicate samples shown as in the main Fig. 3D, panel d1 (A), panel d2 (B), panel e1 (C) and panel e2 (D).

Fig. S8

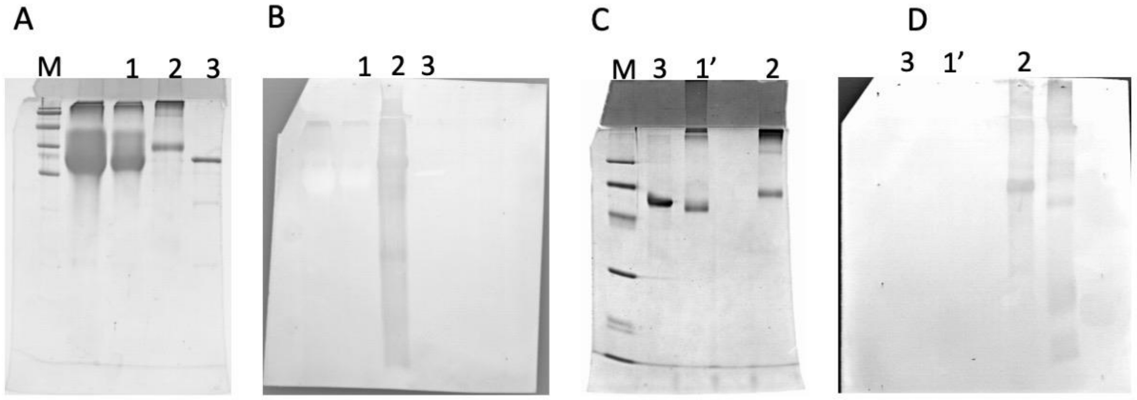

Fig. S9. Original gels and blots of the cropped parts presented in the Fig. 4. The corresponding numbers indicate samples shown as in the main Fig. 4,

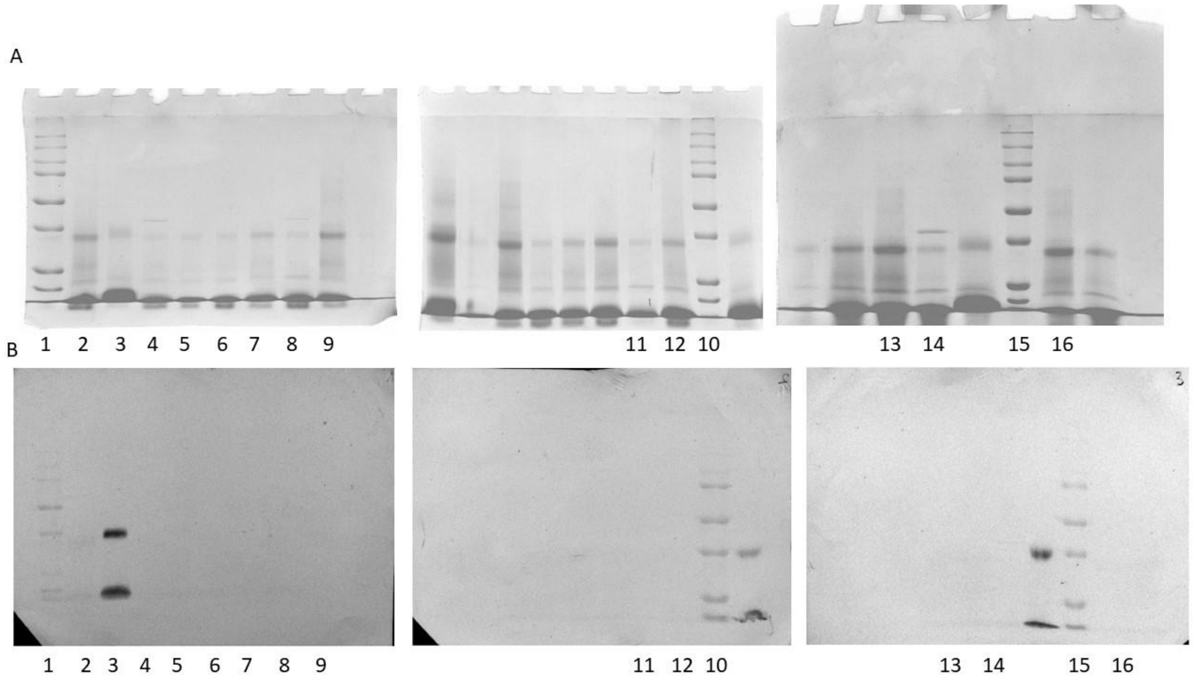

Fig. S10. Original gels and blots of the cropped parts presented in the Fig. S2. The numbers indicate samples shown as in the Supplementary Fig. 2A, panel a1 (A), panel a2 (B), panel e1 (C) and panel C (C).

Fig. S10

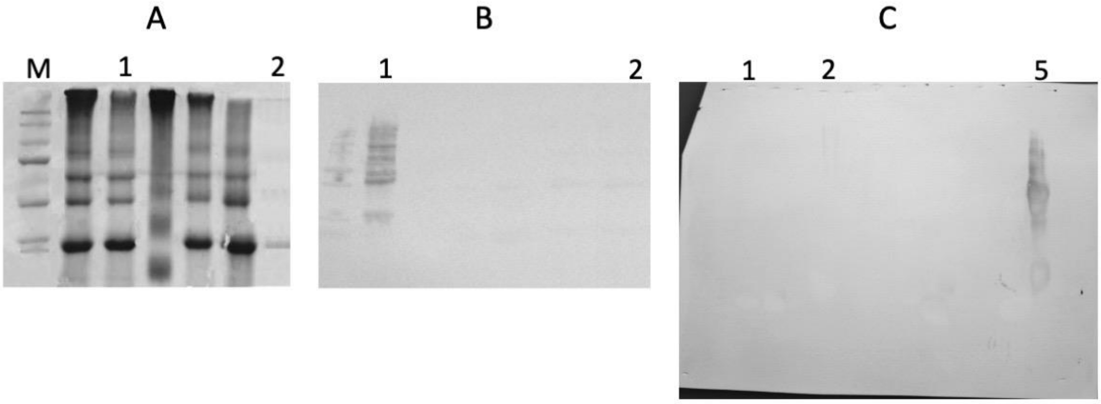

Fig. S11. Original gel and blot of the cropped parts presented in the Fig. S3. The numbers indicate samples shown as in the Supplementary Fig. 3A (A) and Fig. 3B (B).

Fig. S11

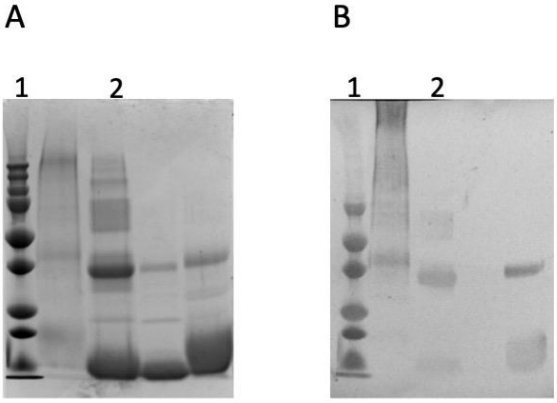

Supplement: Supplementary file 1 — Supplementary Information. [file 41598_2021_82585_MOESM1_ESM.pdf]
